# Effect of tadalafil on blood flow, pain, and function in chronic cold Complex Regional Pain Syndrome: a randomized controlled trial

**DOI:** 10.1186/1471-2474-9-143

**Published:** 2008-10-20

**Authors:** George Groeneweg, Frank JPM Huygen, Sjoerd P Niehof, Feikje Wesseldijk, Johannes BJ Bussmann, Fabienne C Schasfoort, Dirk L Stronks, Freek J Zijlstra

**Affiliations:** 1 Department of Anesthesiology, subdivision Pain Treatment Centre, Erasmus MC, Rotterdam, the Netherlands; 2 Department of Anesthesiology, Erasmus MC, Rotterdam, the Netherlands; 3 Department of Rehabilitation Medicine, Erasmus MC, Rotterdam, the Netherlands, Erasmus MC, Rotterdam, the Netherlands

## Abstract

**Background:**

This double-blind, randomized, controlled trial investigated the effect of the phosphodiesterase-5 inhibitor tadalafil on the microcirculation in patients with cold Complex Regional Pain Syndrome (CRPS) in one lower extremity.

**Methods:**

Twenty-four patients received 20 mg tadalafil or placebo daily for 12 weeks. The patients also participated in a physical therapy program. The primary outcome measure was temperature difference between the CRPS side and the contralateral side, determined by measuring the skin temperature with videothermography. Secondary outcomes were: pain measured on a Visual Analogue Scale, muscle force measured with a MicroFet 2 dynamometer, and level of activity measured with an Activity Monitor (AM) and walking tests.

**Results:**

At the end of the study period, the temperature asymmetry was not significantly reduced in the tadalafil group compared with the placebo group, but there was a significant and clinically relevant reduction of pain in the tadalafil group. Muscle force improved in both treatment groups and the AM revealed small, non-significant improvements in time spent standing, walking, and the number of short walking periods.

**Conclusion:**

Tadalafil may be a promising new treatment for patients that have chronic cold CRPS due to endothelial dysfunction, and deserves further investigation.

**Trial Registration:**

The registration number in the Dutch Trial Register is ISRCTN60226869.

## Background

Complex regional pain syndrome (CRPS) is a painful disorder that typically occurs as a complication of surgery or trauma. There are two types of CRPS: in type 1 no overt nerve lesion is detectable, and in type II a nerve lesion is present [1,2]. Diagnosis is based mainly on consensus-derived clinical criteria [3,4]. In this study we included only patients with CRPS type 1. The main characteristics of CRPS are continuous pain, sensory disturbances, marked changes in tissue blood flow and skin surface temperature, edema, sweating, movement disorders, and trophic changes of the skin. The severity of the symptoms is often disproportional to the initial event [5,6].

During the chronic stage of the disease, inflammatory signs give way to atrophy, reduced regional blood flow, and consequently, reduced skin temperature [7,8]. Impaired microcirculation causes vasoconstriction [9], tissue hypoxia [8], and metabolic tissue acidosis [10,11] that in turn affect the nutritive blood flow in superficial and deep tissues [6,12]. A histopathologic study of skin samples in chronic CRPS showed 'numerous abnormal changes in vascular innervation and structure' [13]. The microcirculation is regulated by neural and endothelial factors [14].

The regulatory neural factors were examined by Wasner et al., who induced whole-body temperature changes to study the cutaneous sympathetic vasoconstrictor activity in CRPS. They identified three vascular regulation patterns: the 'warm', the 'intermediate', and the 'cold', distinguished by skin temperature and by the difference in perfusion values between the affected and contralateral limbs [15]. This asymmetry in blood flow and temperature was dynamic and most prominent at a medium to high level of vasoconstrictor activity. It was suggested that in CRPS, unilateral inhibition of sympathetic vasoconstrictor neurons led to a warmer affected limb in the acute stage, but secondary changes in neurovascular transmission, namely supersensitivity to circulating catecholamines and the increase of alpha-1 adrenoceptors, would lead to vaso-constriction and cold skin in the chronic stage of the disease [15].

Schattschneider et al. investigated the abilities of acetylcholine and sodium nitroprusside to induce endothelium-dependent and endothelium-independent vasodilation, respectively, in patients with CRPS. They concluded that endothelial function was impaired in chronic cold CRPS [14].

The endothelium modulates vascular tone by releasing endothelium-derived vasodilators including nitric oxide (NO), prostacyclin, bradykinin, and endothelium-derived hyperpolarizing factor. In addition, a number of biochemical and physical stimuli cause the release of vaso-constrictors, including endothelin-1 (ET-1) and angiotensin II [16]. Within the vascular smooth muscle cell, NO activates a soluble guanylyl cyclase that elevates the intracellular concentration of cyclic guanosine monophosphate (cGMP). Cyclic GMP in turn activates a specific protein kinase that phosphorylates proteins and ion channels; this results in the opening of potassium channels and hyperpolarization of the muscle cell membrane, sequestration of intracellular calcium by the endoplasmic reticulum, and blocking of calcium influx by the inhibition of calcium channels. The consequence is a drop in cytosolic calcium concentrations and relaxation of the smooth muscle that causes vasodilation [17].

Cyclic GMP is hydrolyzed to GMP by phosphodiesterase type 5 (PDE-5). The inhibition of PDE-5 leads to an increase in the intracellular cGMP concentration. The PDE-5-inhibitor, tadalafil is an effective treatment for erectile dysfunction (ED) [17], and has also been described in the flow-mediated dilation of the brachial artery [18], the nail fold capillary bed of patients with ED [19], and in pulmonary arterial hypertension [20].

The aim of this double-blind, placebo-controlled, randomized clinical trial was to determine whether the PDE-5 inhibitor, tadalafil could improve regional blood flow in the involved extremity of patients with cold type CRPS, and whether this would reduce pain and improve function.

## Methods

### Study subjects, design, and protocol

This double-blind, randomized, placebo-controlled study included 24 patients with cold CRPS. Patient inclusion took place from June 2005 to June 2007 and the data set was completed in September 2007. Potential patients were selected from outpatients of Erasmus Medical Center (MC), from patients that responded to announcements in the Dutch CRPS Patients Association magazine and website, and from patients referred by anesthesiologists at neighboring hospitals. Eligible candidates were invited to visit our outpatient clinic and the pain clinicians FW, FJPMH, and MVM selected patients with stable cold CRPS according to the criteria described by Harden and Bruehl [3]. Additional inclusion criteria were: age between 18 and 60 years old and CRPS limited to one lower extremity. Patients had to be able to stand on the affected leg and walk at least a few steps. Patients with cardiovascular or neurovascular diseases and patients that were hypersensitive to nitrates were excluded.

The sample size calculation was based on the use of the MANOVA repeated measures design on the primary outcome measure temperature difference. An effect size of 0.6 was used, an α of 0.05, and a power of 0.8 (1-β). The total sample size computed by this method was 24 (12 in each group).

Randomization was performed by the Erasmus MC pharmacy according to the research policy of the Erasmus MC, using a computerized randomization list. Patients, researchers, and physicians were blind to the intervention administered; only the pharmacist had the allocation code.

Patients were randomized (12 per group) to receive either tadalafil or a placebo over a 12-week period. Patients in the tadalafil group started with 10 mg daily during 4 weeks and then took 20 mg daily for another 8 weeks. This titration schedule was advised in order to avoid tolerance effects. Patients in the placebo group also followed this schedule of titration. Because, in the Netherlands, the use of Tadalafil has not yet been approved for CRPS, the study medication was stopped after the study period and the patients were seen by FJPMH to discuss further conventional treatment. All patients participated in a modified version of the physiotherapy program described by Kemler et al. [20], which is based on a graded activity approach intended to improve function, strength, and mobility of the affected extremity. The patients performed daily exercises at home, which was instructed and supervised by a local physiotherapist during one therapy session per week. The therapists received written instructions, filled in compliance reports and received feedback by telephone and email by the first author.

### Outcome measures

To determine the effects of tadalafil on the microcirculation, the primary outcome measure was the temperature difference between the CRPS affected and the contralateral limbs. The secondary outcome measures were pain, muscle force, and activity. Outcome measures were assessed at the start and end of the study.

Temperature was measured using video thermography. In chronic cold CRPS, a reduction in local skin temperature is directly related to diminished tissue blood distribution [21]. Video thermography was shown to be an effective method for monitoring near-surface blood flow in the limbs [22,23]. The skin temperatures of both feet were registered with a computer-assisted infrared thermograph (ThermaCAM SC2000, Flir Systems, Berchem, Belgium) following a standard protocol [24]. To compare thermographic images of the feet, the means and difference in temperature (°C) were calculated as described previously [22]. Under normal conditions, the thermal asymmetry between opposite sides of the body is very small; in healthy subjects, the degree of thermal asymmetry was less than 1% (< 0.25°C) when measured with computerized thermography [22,25,26].

Pain is often described as the most prominent feature of CRPS [27]. The patients were instructed to record daily pain intensity, concurrent medication, and adverse events in a diary. The pain intensity was rated according to a Visual Analogue Scale (VAS) scale (0–100 mm). The actual pain score was recorded three times daily (0800, 1200 and 2000 h) during the week before the first and last hospital visits; the average scores for each of those weeks were used in the analyses.

Function was evaluated with muscle force tests, walking capacity tests, and measurements of performance. Muscle force of the flexors and extensors of the knee and foot was measured using a MicroFet 2 dynamometer (Hoggan Health Industries Inc, West Jordan, UT, USA) as described by Bohannon and Andrews [28-30], using the 'break' method [28]. The mean value of three measurements was calculated and given in units of Newtons (N).

A ten meter walking test and a two minute walking test were performed to determine the walking ability [31-33]. The first test was performed three times, at both comfortable and maximum walking speeds, and timed with a stopwatch. The two minute walking test measured the non-stop walking distance in meters.

To determine possible differences between perceived and actual changes in activity levels, patients wore an Activity Monitor (AM) and answered a questionnaire. The AM was used to measure actually performed physical activity [34,35]. The device is based on ambulatory accelerometry and enables objective determination of activity in different postures (lying, sitting and standing) and motions (walking and general movement) during everyday functions. It is increasingly used in research involving a variety of patient groups, including acute [36] and chronic [37,38] CRPS patients. The signal analysis and output were described previously [34,35,39].

After all other measurements were completed, the AM was fitted on each patient to measure activity over a 24-h period in the hospital at the start and the end of the study. The patients were instructed to continue their normal everyday activities while wearing the AM with the exception of swimming, bathing, or showering. The following AM outcome measures were calculated: the percentage of time spent standing, walking, or in an upright position, and the number of short walking periods (< 10 s). In these measures a higher value represented better performance.

Perceived activity limitations were measured with a questionnaire developed by the Dutch Measuring Mobility Study Group that consisted of 35 questions [40]. In the questionnaire a lower value represented better performance.

Blood pressure and pulse frequency were also recorded at every visit.

### Statistical analysis

Differences in patient characteristics between the treatment groups were analyzed with the Mann-Whitney U test, Fisher's Exact test, and the chi square test, as appropriate.

A multivariate repeated-measures design was used to analyze differences in mean parameters between the treatment groups, and over time (between the starting and ending values), with group (tadalafil or placebo) and time (start and end) as independent variables. In addition, we analyzed the interaction between treatment and time. This method of analysis was used despite the skewed distribution of the outcome measures, due to the robustness of the analysis of variance [41]. Alpha was set to the conventional level of 0.05. Analyses were performed with the Statistical Package for the Social Sciences, version 14.02 (SPSS Inc., Chicago, IL, U.S.A).

### Statement of compliance with ethical regulations

The Medical Ethics Committee of the Erasmus MC approved the study protocol (MEC 2004-159). The research was performed in accordance with the Declaration of Helsinki (2000) of the World Medical Association, and written informed consent was obtained from all participants.

The registration number in the Dutch Trial Register is ISRCTN60226869.

## Results

**Figure 1:**
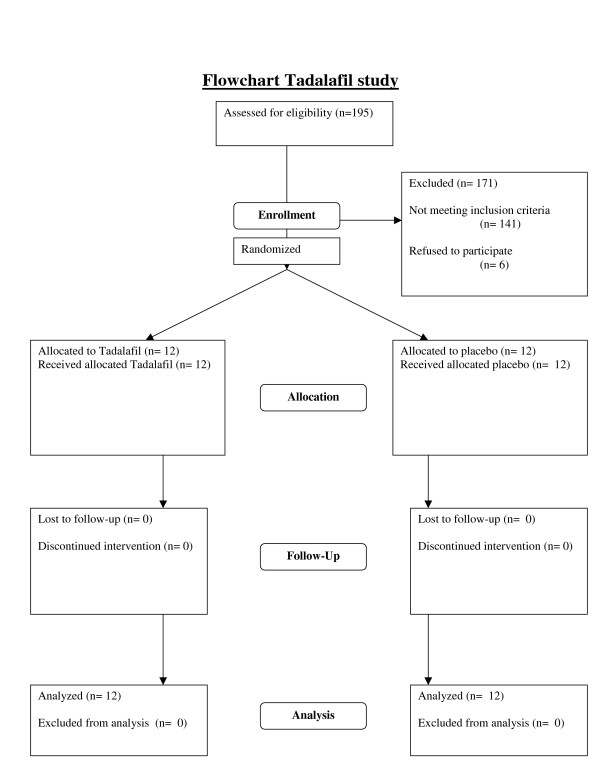
Flowchart Talafil study.

As the flowchart in figure 1 shows, twenty-four patients participated and completed the study. The baseline characteristics of the patients assigned to the tadalafil and placebo groups are shown in Table 1. There were no significant differences in age, gender, duration of disease, or smoking habit between the two experimental groups.

**Table 1:** Patient characteristics

	**tadalafil**	**placebo**	***P-value***
*n*	12	12	
Age, years	39.8 ± 13.1	36.5 ± 10.6	0.56 ^1^
Gender (female/male)	9/3	11/1	0.29 ^2^
Duration of CRPS, months	37.1 ± 24.5	55.7 ± 52.3	0.60 ^1^
Smoking (yes/ex-smoker/no)	6/1/5	4/2/6	0.66 ^3^
Temperature of CRPS foot, °C	26.8 ± 2.8	28.3 ± 4.7	0.60 ^1^
Temperature of contra lateral foot, °C	28.5 ± 2.8	29.9 ± 3.3	0.27 ^1^
Difference in temperature, °C	1.7 ± 0.77	1.7 ± 2.6	0.41^1^

^1 ^Mann Whitney U test; ^2 ^Fisher's Exact test; ^3 ^χ^2^

test Values are the mean ± SD unless otherwise indicated

The results of the videothermographic measurements are given in Table 2. Although the temperature difference appeared to be reduced in the tadalafil group and increased in the placebo group, the probability value was only 0.37; thus we cannot exclude that these effects were due to sampling error. However, there was a significant reduction in pain intensity after tadalafil treatment, as indicated by a 15% reduction of the VAS (from 61.3 to 52.3) in the tadalafil group. In contrast, the pain intensity in the placebo group remained unchanged. Since the outcome data were only assessed at the start and end of the study, we cannot provide information on the time course of these changes. We do know, however, from the remarks of the patients, that most changes in temperature and pain appeared to take place after 4 weeks, after the medication had been doubled from 10 to 20 mg daily.

**Table 2:** Temperature and Pain.

	**tadalafil**		**placebo**			
	**start**	**end**	**start**	**end**	**P_t_**	**P_gt_**
**Temperature difference (in °C)**	1.71 ± 0.77	1.09 ± 1.6	1.65 ± 2.6	1.77 ± 1.9	*0.54*	*0.37*
						
**Pain intensity VAS (0–100 mm)**	61.3 ± 14.1	52.3 ± 19.1	57.0 ± 12.1	56.5 ± 10.8	0.03*	0.04*

The difference in temperature (°C) between the two feet, measured with videothermography. The Visual Analogue Scale (VAS) was used to measure pain intensity three times daily during the week preceding the hospital visits. The start and end times were when treatment started at t = 0 and ended at t = 12 weeks.

P_t _= *P*-value for differences over time; P_gt _= *P*-value for interaction Group*Time

Table 3 shows the difference in muscle strength between the contralateral and CRPS affected sides. We observed significant improvement over time in both groups for knee extension, but we failed to find significant differences between treatment groups.

**Table 3:** Differences in muscle strength.

	**tadalafil**		**placebo**			
	**start**	**end**	**start**	**end**	**P_t_**	**P_gt_**
**Knee extension**	64.2N (64.8%)	56.7N (70.3%)	97.7N (45.2%)	80.3N (58.9%)	0.01*	*0.05*
						
**Knee flexion**	52.0N (66.1%)	45.2N (70.7%)	70.4N (52.9%)	43.5N (71.8%)	*0.12*	*0.08*
						
**Foot dorsal flexion**	79.6N (53.6%)	84.3N (51.0%)	102.5N (39.5%)	81.4N (52.1%)	*0.40*	*0.28*
						
**Foot plantar flexion**	93.0N (56.4%)	100.2N (57.0%)	134.6N (42.4%)	76.1N (64.1%)	*0.05*	*0.34*

Results of the muscle force tests measured with the MicroFet 2 Dynamometer. Values indicate the differences in muscle strength between the CRPS affected and contralateral sides. All values are given in units of Newtons (percent difference between the two sides). The start and end times were when treatment started at t = 0 and ended at t = 12 weeks.

P_t _= *P*-value for differences over time; P_gt _= *P*-value for interaction Group*Time

Table 4 shows the outcome measures for the actually performed activity. The results of the Activity Monitor indicated that the intervention did not significantly affect patient activity levels. This result was corroborated by the lack of changes detected in either the ten meter walking tests or the questionnaire (Table 5). However, there was a significant change over time in both treatment groups in the two minute walking test, though the difference between groups was insignificant.

**Table 4:** Activity limitations determined using the AM.

	**tadalafil**		**placebo**			
	**start**	**end**	**start**	**end**	**P_t_**	**P_gt_**
**% Time Standing**	8.3 ± 2.8	10.7 ± 6.1	10.5 ± 4.3	10.3 ± 3.3	*0.25*	*0.19*
						
**% Time Walking**	4.5 ± 2.2	5.3 ± 2.8	5.6 ± 2.9	6.5 ± 3.8	*0.15*	*0.88*
						
**% Time Upright**	12.8 ± 4.5	16.0 ± 8.3	16.1 ± 7.0	16.9 ± 6.9	*0.18*	*0.40*
						
**Number of Walking periods < 10 s**	119.9 ± 50.8	138.7 ± 67.9	141.7 ± 81.8	146.4 ± 58.8	*0.40*	*0.61*

The Activity Monitor measurements were taken over a 24-h period, and are given as mean ± SD. In all these measurements a higher value indicates better performance. The start and end times were when treatment started at t = 0 and ended at t = 12 weeks.

P_t _= *P*-value for differences over time; P_gt _= *P*-value for interaction Group*Time

**Table 5:** Walking tests.

	**tadalafil**		**placebo**			
	**start**	**end**	**start**	**end**	**P_t_**	**P_gt_**

**10 m, comfortable speed **(time in seconds)	16.2 ± 13.0	16.3 ± 14.1	15.6 ± 8.2	13.2 ± 5.9	*0.36*	*0.34*
**10 m, maximal speed **(time in seconds)	9.8 ± 4.4	9.2 ± 4.5	7.9 ± 1.9	8.6 ± 2.8	*0.76*	*0.06*
**2 minute walking test **(distance in m)	106.9 ± 34.9	119.6 ± 46.3	92.5 ± 30.4	111.8 ± 32.6	0.01*	*0.49*
						
**Walking questionnaire scores**	27.5 ± 6.9	25.3 ± 10.6	27.6 ± 6.0	28.1 ± 5.7	*0.44*	*0.22*

Results from the walking tests. The patients performed the 10 meter walking test three times at a comfortable speed, and again three times at maximal speed. The mean of the three tests is given ± SD. For the two minute walking test the patients had to walk for 2 minutes, and the distance in meters is given. For these three walking tests, a higher value indicates better performance, but for the questionnaire improvement is indicated by a lower value. The start and end times were when treatment started at t = 0 and ended at t = 12 weeks.

P_t _= *P*-value for differences over time; P_gt _= *P*-value for interaction Group*Time

Systolic blood pressure was 134 (± 10) mmHg at the start in the placebo group, and 130 (± 16) mmHg at the end of the trial. In the tadalafil group the initial systolic blood pressure was 142 (± 18) mmHg and at the end of the study it was 140 (± 19) mmHg. There was no significant difference between treatment groups, and no changes were observed in diastolic blood pressure.

Most patients in the tadalafil group reported a warmer affected extremity, sometimes coupled with an itching sensation. Two patients complained about painful muscles in their whole body during the first few weeks of the trial. Several patients in both groups experienced the measurements as very tiresome, and felt exhausted afterwards, however, there were no severe adverse events.

## Discussion

Although this double-blind, randomised, placebo-controlled study did not show a significant improvement in blood flow in the extremities as observed by video thermography, we found a significant and clinically relevant reduction of pain in patients with chronic cold CRPS.

It has been repeatedly suggested that the temperature asymmetry between extremities is an important diagnostic measure for CRPS [42,43]. In this trial, we found a considerable temperature asymmetry in all patients at baseline that was reduced in the tadalafil group, and slightly increased in the placebo group after the treatment period. Although our analysis suggested these treatment effects were insignificant, we recommend caution in interpreting these results because the study had low statistical power as a result of the large variance and relatively low covariance between time points. The observed statistical power of the treatment × time factor was only 0.14 (alpha = 0.05). In contrary to the placebo group, most patients in the tadalafil group were very satisfied with the treatment, claiming that the leg was much warmer under almost all circumstances. Therefore, the treatment effects may be clinically relevant. Although the primary indication for PDE-5 inhibitors is erectile dysfunction (ED), prolonged treatment with tadalafil has a vasodilative effect that has also been described for other arteries [18-20]. However, one study in patients with Raynaud's phenomenon (RP) showed that a single dose did not improve digital blood flow at baseline, increase the response to heating, or attenuate cold-induced vasoconstriction [44]. However, a review that included several reports found that prolonged treatment with tadalafil was associated with improved microcirculation, symptomatic relief, and ulcer healing in patients with secondary RP [45].

Our results are in accordance with the latter conclusions. We observed a reduction in the temperature asymmetry that was most likely caused by restored microcirculation. The variation in temperature data may be an indication that in some patients other, possibly central, thermo-regulatory mechanisms may have interfered with the peripheral blood flow.

Our patient population is too heavily weighted towards women. The usual ratio is 4:1 [46,47], and we included 20 women and 4 men. Although it has been suggested, that the influence of hormonal etiological factors may be involved in the pathogeneses of CRPS [47], there are no indications, that these factors still play a role in the vascular alterations in the chronic CRPS.

Pain, which is the most important parameter of CRPS, was significantly reduced compared to placebo. However, in pain studies a 30% increase is usually considered meaningful, and 50% robust. Since we found a mere reduction of 15%, this should be interpreted with care. Pain in CRPS may be due to a neuropathic cause [13,48], there may be sympathetically maintained pain [49], or it could be caused by local ischemia [8,50], perhaps even in the same patient. It seems likely, that the present reduction in pain was achieved by an improvement in local ischemia, leaving the other causes unchanged. On the other hand, an increased physical activity could consequently result in an increased awareness of pain. This needs further investigation.

We also observed a small, but insignificant improvement in muscle force over time. This increase might have resulted from physical therapy and the home exercise program, or it could have been due to a learning effect after repeated muscle force testing. In both groups we observed a slight reduction in the asymmetry in muscle force between the CRPS affected and contralateral sides. The asymmetry was largest in the foot dorsal flexion. However, foot dorsal flexion was also partly influenced by the pain arising from the pressure of the measurement device on the plantar side of the foot; this was the most painful area in most patients. Because these improvements were observed in both groups, muscle force was not apparently influenced by the general pain reduction in the tadalafil group.

During the trial, most patients in the tadalafil group experienced a large improvement in walking ability. Some reported that they were able to leave their crutches at home when walking short distances, even outdoors. However, the results of the walking tests did not reflect this improvement. We found only a small, non-significant progression in the ten meter walking tests. A closer evaluation revealed that some of the improved patients were walking with two crutches at high-speed at the start of the trial, and walked without crutches at the end of the trial, but at the cost of a slower walking speed. Thus, the improvement was not expressed in speed, but in quality of walking. We found significant increases in the two minute walking test in both treatment groups, and an indication of improvement in the walking questionnaire. These findings were supported by the results from the activity monitoring. Although the improvements were insignificant, there were small increases in standing, walking, and the number of short walking periods.

Although we assumed we had recruited a homogeneous study population with chronic cold CRPS, not all patients reacted similarly to the intervention. Most patients responded very well to tadalafil, and reported a warmer extremity with a reduction of pain, but two patients experienced no improvement at all, and the CRPS side remained up to 4 °C colder than the unaffected side. This lack of responce was most likely due to the presence of other central or peripheral factors that contributed to the disturbed vasodynamics in chronic cold CRPS, factors that were not influenced by cGMP stimulation through PDE-5 inhibitors. On the other hand, two patients in the placebo group experienced an almost complete recovery from pain and impairment. This might have been due to the effects of physical therapy, or a combination of the effects of the therapy and extra attention they received while performing tests as a trial participant. In either case, these improvements indicate that a program directed at improving function may be very effective for patients with chronic cold CRPS, even in cases that have lasted more than four years.

This trial was limited to a period of 12 weeks. Little is known about the effects of using PDE-5 inhibitors over a longer period. In patients who experienced reduced pain as a result of tadalafil, a remarkable improvement in function was observed. The use of crutches was reduced and the number of short walking periods increased. However, the perceived improvements were small, as reported in the walking ability questionnaire. Thus, it may take a longer period of time for patients to acknowledge the extent of improvements. We speculate that over time, with tadalafil patients may acquire an activity pattern closer to normal and improvements in function due to the reduction of pain and the subsequent reduction in fear-avoidance, which has been described as one of the limiting factors in CRPS [51].

It has been suggested that CRPS is primarily a disease of the central nervous system [52]. In any case it is a multifaceted disorder, and any effort to treat only one facet is bound to result in failure [53]. However, in this trial we have solely concentrated on the question, whether the inhibition of PDE-5 could improve the blood flow in CRPS. Future investigations should include a larger study group of patients with chronic cold CRPS and an effort should be made to select only patients with endothelial dysfunction. A longer trial period would be required to investigate whether the improvements last. It would also be interesting to investigate whether this newly gained improvement in function, supported by an activity program if necessary, would be sufficient to sustain further progression in activity.

## Conclusion

Tadalafil may be a promising new treatment for patients that have cold CRPS due to endothelial dysfunction. The PDE-5 inhibitor produced a clinically relevant reduction in the asymmetry in temperature between the affected and unaffected feet in CRPS, most likely due to the restoration of blood flow in the affected extremity. We also observed a significant reduction of pain compared to placebo, likely due to an increase in vasodilation and the abolishment of ischemia. Future investigations should focus on the long-term effects of tadalafil on improvements in function.

## Competing interests

The authors declare that they have no competing interests.

## Authors' contributions

JGG carried out the study, performed measurements and drafted the manuscript, FJPMH assisted in the patient inclusion and helped to draft the manuscript, SPN performed the videothermographic recordings and calculated the temperature values, FW assisted in the measurements and the patient inclusion, JBJB participated in the design of the study and the Activity Monitor measurements, FCS calculated the Activity Monitor data and helped to draft the manuscript, DLS participated in the design of the study and performed the statistical analysis, and FJZ designed and supervised the study and helped to draft the manuscript. All authors read and approved the final manuscript.

## Pre-publication history

The pre-publication history for this paper can be accessed here:


